# Molecular Characterization of African Swine Fever Viruses Circulating in Can Tho City, Vietnam

**DOI:** 10.1155/2023/8992302

**Published:** 2023-02-02

**Authors:** Nguyen Duc Hien, Le Trung Hoang, To My Quyen, Nguyen Phuc Khanh, Lam Thanh Nguyen

**Affiliations:** ^1^Faculty of Veterinary Medicine, College of Agriculture, Can Tho University, Campus II, 3/2 Street, Ninh Kieu, Can Tho, Vietnam; ^2^Can Tho Sub-Department of Animal Health, Ministry of Agriculture and Rural Development, 30/4 Street, Ninh Kieu, Can Tho, Vietnam

## Abstract

African swine fever (ASF) is a highly contagious and deadly viral disease in domestic and feral pigs. Since 2018, the disease has spread and caused large socioeconomic consequences to the pig industry in several Asian countries including China, Vietnam, and South Korea. This study aims to determine the genotype, serotype, and genetic variation of representative ASF viruses (ASFV) responsible for the outbreaks in 2019–2022 in Can Tho city, a central administrative province in the Mekong delta, Vietnam. For outbreak investigation, the presence of causative ASFVs was tested using conventional PCR targeting the B646L gene. Subsequently, the amplification and sequencing of the DNA fragments of the putative B646L gene encoding the major capsid protein p72, EP402R gene encoding the viral hemagglutinin CD2-like protein (CD2v), and intergenic region (IGR) between the l73R and I329L genes were performed for molecular characterization. Phylogenetic analyses based on B646L and EP402R genes confirmed that all ASFVs detected in Can Tho city belonged to genotype 2 and serotype 8. In addition, this study revealed that at least two variants of ASFVs, namely, IGR II and IGR III, based on the nucleotide variation of the IGR sequence, cocirculated, and caused outbreaks in Can Tho city. The molecular characterization study provides great significance for understanding the evolution of ASFVs and tracing possible sources of infection in Can Tho and Mekong delta.

## 1. Introduction

African swine fever (ASF) is a highly contagious disease in swine characterized by acute hemorrhagic fever and high mortality. Owing to the high transmissibility and serious socioeconomic consequences of the disease, the World Organization for Animal Health (OIE) classified ASF as a list A notifiable disease (https://www.woah.org/en/what-we-do/animal-health-and-welfare/animal-diseases/old-classification-of-diseases-notifiable-to-the-oie-list-a/). ASF is caused by African swine fever virus (ASFV), which belongs to the *Asfivirus* genus, the only member of the *Asfarviridae* family [1]. The ASFV genome is a linear double-stranded DNA of approximately 170–193 kbp encoding at least 160 open reading frames [2, 3]. Currently, there are 24 genotypes and 8 serotypes that have been reported worldwide based on the B646L gene encoding the major capsid protein p72 and EP402R gene encoding the viral hemagglutinin CD2-like protein (CD2v), respectively [4–6]. Furthermore, differentiation between close strains within each genotype can be determined by the variation of the nucleotide sequences of the partial intergenic region (IGR) located between the I73R and I329L genes [7].

In Vietnam, the first ASF outbreak was reported at a backyard pig farm in the northern province in February 2019 [8]. Since then, serial outbreaks of ASF in domestic pigs have continuously been reported and have become endemic across the country [9]. Recently, several studies have been conducted to determine the genotypes and serotypes of ASFV circulating in Vietnam. All of these studies indicated that ASFVs causing outbreaks also belonged to genotype 2 and serotype 8 as in previous studies, suggesting that these are the most predominant serotype and genotype in Vietnam [9–11]. Furthermore, genetic characterization has been used previously to genetically differentiate the variation between closely related ASFVs based on the analysis of the IGR between the I73R and I329L genes [7]. In Vietnam, IGR I and IGR II variants with three tandem repeat sequences (TRS) were initially reported in the northern areas in 2020 [12], and variant IGR III with four TRS was later found in at least four different provinces of Northern Vietnam during the 2019–2022 outbreaks [9], indicating that multiple variants of genotype II of ASFVs currently circulate in Vietnam.

In Southern Vietnam, limited molecular studies on ASFVs have been conducted. A few published studies showed that ASFVs circulating in this region also belonged to genotype 2 and serotype 8 [13, 14]. However, these studies still provide limited information on the molecular properties and epidemiology of this virus. Therefore, further investigations are needed to provide more insights into the genetic characterization and variation of ASFVs. In this study, Can Tho city, which is considered an epicenter of ASF in the region due to its geographical and socioeconomic importance in the Mekong delta, has been selected as a representative study site for the molecular investigation of ASFVs in the Mekong delta. This study aims to determine the genotype, serotype, and genetic variations of representative ASFVs causing outbreaks in Can Tho city in 2019–2022 based on the sequences of B646L (p72) and EP402R (CD2v) genes and the TRSs between the I73R and I329L genes, respectively.

## 2. Materials and Methods

### 2.1. Study Areas and Sample Collection

**Figure 1:**
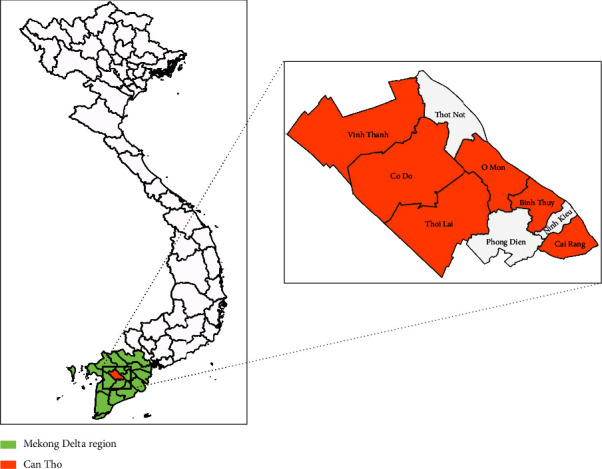
Map of Vietnam and Can Tho city showing locations where samples of ASF outbreaks were collected in this study. The Mekong delta is colored green and the six representative districts in Can Tho city are colored orange.

During 2019–2022, seven representative ASF outbreaks in domestic pigs reported by the provincial Can Tho Sub-Department of Animal Health were selected for this study. An outbreak was defined as at least one blood or tissue sample taken from affected pigs on a farm returning a positive result when tested with ASF real-time PCR. These seven outbreaks occurred in six (out of nine) administrative units of Can Tho that were selected for the purposes of this study, namely, including Binh Thuy, Cai Rang, Co Do, O Mon, Thoi Lai, and Vinh Thanh (Figure 1). Tissue samples including sets of lymph nodes and spleens and/or whole blood were collected from fatal and symptomatic pigs with ASF to detect causative ASFVs and molecular characterization. The details of space–time and types of collected samples are shown in Table 1.

### 2.2. DNA Extraction and PCR Assay

Total viral DNAs were extracted from the collected tissue samples or whole blood using the TopPURE® Tissue Viral Extraction kit and TopPURE® Serum Viral Extraction kit (TBR, Vietnam), respectively, following the recommendation of the manufacturer. Genomic DNA was eluted in 35 *μ*L of elution buffer of the kit and stored at −80°C until further use. Next, the DNA extracts from ASFV-infected samples were used for conventional PCR amplification with three pairs of primers of (i) P72-U/P72-D targeting the B646L gene [4], (ii) ASF_CD2v_ga3611-F/ASF_CD2v_ga4220-R targeting the EP402R gene [15], and (iii) ASF_IGR_I73R_F/ASF_IGR_I73R_R targeting the fragment of IGR located in the I73R and I329L genes [14]. The detail of the primer sequences used in this study is described in Table 2.

The PCR amplification reactions were performed in a 22 *μ*L volume, containing 11 *μ*L MyTaq™ master mix (Bioline, USA), 3 *μ*L template DNA, 1 *μ*L of 10 *μ*M each primer (forward and reverse), and 6 *μ*L ultrapure water. The thermocycling condition for PCR detection of ASFV followed 40 cycles of denaturation at 98°C for 10 s and extension at 72°C for 60 s with a 7-min elongation at 72°C after an initial denaturation step at 95°C for 5 min. The following thermal cycling program was used with a moderate modification in annealing temperature from 55°C to 58°C based on the melting temperature of the primers indicated in Table 2. The products of PCR amplification were electrophoretically analyzed on a 1.5% agarose gel containing ethidium bromide (1 *μ*g/mL) in 1 × Tris-acetate-EDTA (TAE) buffer before visualization and imaging using UV transillumination (BIO-RAD, USA).

### 2.3. Nucleotide Sequencing and Phylogenetic Analysis

Three amplicons of B646L (478 bp), EP402R (850 bp), and IGR (651 bp) were purified from the PCR products using the TopPURE® PCR/Gel ADN purification kit (TBR, Vietnam). The DNA purified products were sent to Genlab Co., Ltd., Vietnam, for Sanger sequencing. Sanger sequencing that used the ABI Prism BigDye™ Terminator v1.1 cycle sequencing kit was conducted using an by ABI PRISM 3500 × *l* Genetic Analyzer. The nucleotide sequences of seven ASFVs derived from three defined gene fragments were analyzed with BioEdit® Sequence Alignment Editor version 7.1.9. The basic local alignment search tool was used to verify the similarities of the sequences obtained in this study with reference sequences retrieved from the GenBank. The Clustal W process in MEGA 7.0 was used for sequence alignment, and nucleotide positions after alignment were numbered according to the numbering scheme of Georgia 2007/1 (NC_044959.2) throughout the text. A maximum-likelihood tree for each B646L and EP402R gene sequence was constructed using MEGA 7.0 with a resampling process of 1000 replicates to determine the genotype and serotype of ASFVs [4, 6, 16].

## 3. Results

### 3.1. Laboratory Confirmation of ASFV

**Figure 2:**
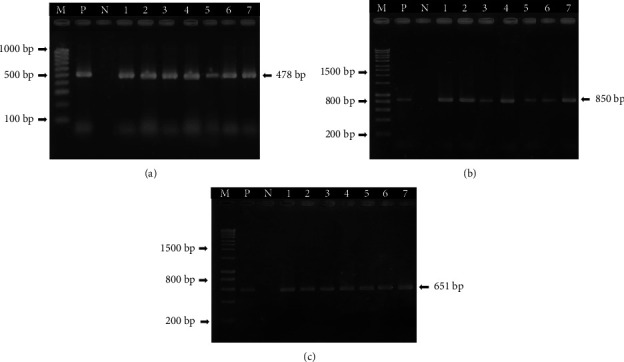
(a) PCR amplification of the B646L gene encoding p72 protein, (b) the EP402R gene encoding CD2v protein, and (c) the IGR between I73R and I329L genes. Lane M 1 Kb molecular weight DNA marker, lane P a positive control, and lane N is the negative control. Lane 1: ASF/Binh Thuy/2019, lane 2: ASF/Thoi Lai/2019, lane 3: ASF/Co Do/2021, lane 4: ASF/Cai Rang/2021, lane 5: ASF/O Mon/2021, lane 6: ASF/Vinh Thanh/2021, lane 7: ASF/Cai Rang/2022. Lanes 1, 2, 3, 4, 5, 6, and 7 are selected positive samples with band size of an approximately 478 bp, 850 bp, 651 bp, respectively.

During the period of 2019–2022, seven representative outbreaks of ASF in domestic pigs were subjected to this molecular investigation. Tissue samples and/or whole blood collected from fatal and symptomatic pigs in six representative districts of Can Tho were tested for the presence of ASF viral DNA using conventional PCR. Diagnostic confirmation was conducted based on the ASFV-specific primers, B646L gene encoding for protein p72, EP402R gene encoding for protein CD2v, and IGR between the I73R and I329L genes.  Figure 2 shows the PCR results with evident amplicons of the expected sizes that confirmed the presence of ASFVs in all collected samples.

### 3.2. Genotyping ASFVs Using the Partial B646L (p72) Gene

**Figure 3:**
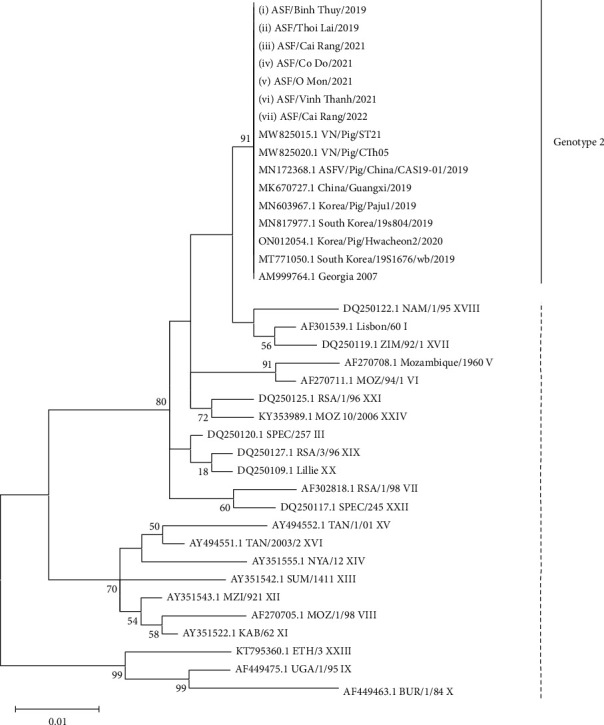
A maximum-likelihood phylogenetic tree based on the complete sequences of B646L gene (encoding for serotype-specific proteins p72) of ASFV. The Kimura 2-parameter model was used for construction of the phylogenetic tree using MEGA 7.0 software. Numbers along branches indicate bootstrap values >70% (1,000 replicates). The bars and numbers on the right indicate genotypes of ASFV. Black circles indicate the ASFVs detected in this study causing an outbreak in Can Tho during 2019–2022.

To determine the genotype and genetic relationship of the ASFVs in this study and other reference ASFVs previously deposited in the GenBank, a phylogenetic tree was constructed based on the partial sequences of the B646L (p72) gene. The phylogenetic tree indicated that all Can Tho ASFVs were grouped into genotype 2, together with ASFVs previously detected in Vietnam and China (Figure 3).

### 3.3. Serotyping ASFV Using the Partial EP402R (CD2v) Gene

**Figure 4:**
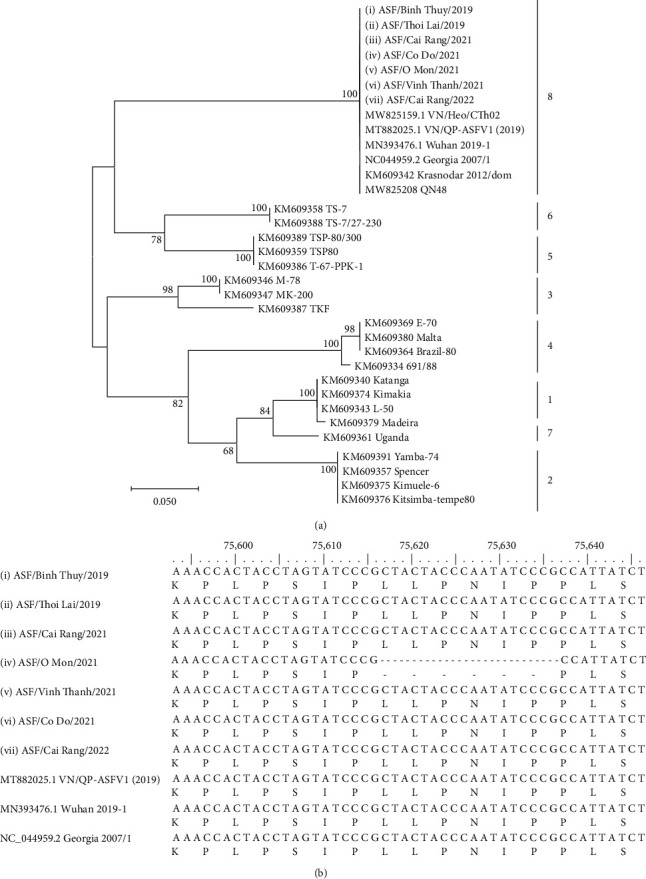
(a) A maximum likelihood phylogenetic tree based on the complete sequences of EP402R gene (encoding for serotype-specific proteins CD2v) of ASFV. The Kimura 2-parameter model was used for construction of the phylogenetic tree using MEGA 7.0 software. Numbers along branches indicate bootstrap values >70% (1,000 replicates). The bars and numbers on the right indicate serotypes of ASFV. Black circles indicate the ASFV detected in this study causing an outbreak in Can Tho during 2019–2022. (b) Alignment of partial sequences in the EP402R of seven ASFVs in this study and other reference ASFVs. Black circles indicate the ASFVs detected in this study causing an outbreak in Can Tho during 2019–2022.

To determine the serotype of the representative ASFVs, another phylogenetic tree based on the partial sequence of the EP402R (CD2v) gene was constructed. The result indicated that Can Tho ASFVs clustered into serotype 8 is identical to the serotype of ASFVs detected in Vietnam, China, and Korea (Figure 4(a)). Noteworthily, the sequence alignment of the EP402R gene detected an 18-bp nucleotide deletion of “CTACTACCCAATATCCCG” that results in six-amino-acid deletion “LLPNIP” in the CD2v of ASF/VN/CanTho-OM/2021 (Figure 4(b)). The short mutation sequence in the EP402R gene was confirmed as unique because there was no homology to other sequences previously deposited in the GenBank (data not shown).

### 3.4. Determining Genetic Variation Based on IGR between the I73R and I329L Genes

**Figure 5:**
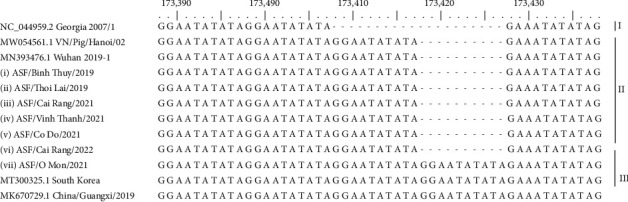
Alignment of partial nucleotide sequences of the IGR between l73R and I329L of ASFV in Can Tho city. Six ASFVs indicating a 20-bp insertion in the TRS while only one strain insertion a 10-bp in the TRS as compared to other sequences deposited previously in the GenBank. Black circles indicate the ASFVs detected in this study causing an outbreak in Can Tho during 2019–2022.

To determine the genetic variation among identified ASFVs, comparative DNA alignment of the sequences in the IGR between the I73R and I329L genes of ASFVs obtained in this study and reference ASFVs was performed. The results showed that six out of seven ASFVs causing outbreaks in Can Tho in 2019–2022 belonged to the IGR II variant that contains three TRS insertions (Figure 5). Only one strain, ASF/VN/CanTho-OM/2021, was determined to belong to the IGR III variant containing four TRS insertions, which is identical to the ASFVs previously detected in China (MK670729.1) and South Korea (MT300325.1).

## 4. Discussion

ASF has been endemic in Can Tho and it posed a major obstacle to the development of the pig industry in the region. A total of 2,377 ASF outbreaks have been reported across nine districts in this region in early 2019 [17]. To the best of our knowledge, this study is the first report on the genetic characterization of representative ASFVs that caused outbreaks in Can Tho city in 2019–2022.

A phylogenetic tree based on the putative gene B646L (p72) was constructed to determine the genotype of ASFVs. Our result showed that all ASFVs in this study belonged to genotype 2 (Figure 3). This result is in agreement with several previous studies that have reported that only ASFVs belonging to genotype 2 have been in circulation in Vietnam [10, 11]. This finding indicates that ASFV infections that were initially described only in Northern Vietnam (north central and the Red River delta regions) may have spread to the southern region through movement, trade of pigs, and pig products despite the official control strategies in those regions. Since then, it has been confirmed that genotype 2 is the most prevalent genotype in the southern region since the molecular characterization of the circulating ASFVs originating from local outbreaks was serially grouped into genotype 2.

In addition, to determine the serotype of the studied viruses, further genetic analyses of seven ASFVs in Can Tho based on the phylogenetic tree of the EP402R (CD2v) gene were performed. The EP402R phylogenetic analysis showed that the ASFVs detected in Can Tho together with other reference strains from Vietnam, China, and South Korea are a member of serogroup 8 (Figure 4(a)). However, the sequence alignment of the EP402R gene detected a mutation with the absence of an 18-bp nucleotide (six-amino-acid deletion “LLPNIP”) in a single strain, ASF/VN/CanTho-OM/2021, out of the seven representative ASFVs (Figure 4(b)). Importantly, the short mutation sequence was confirmed as a unique mutant because there was no homology to other sequences of GenBank-deposited ASFVs. The effects of this mutation on the molecular mechanisms of the function of this viral protein are the main issue of further extensive investigations.

Although only ASFV genotype 2 and serotype 8 were identified in Can Tho, our analysis of the TRS in the IGR indicates more diversity among these isolates. It has been reported that the analysis of the IGR between the I73R and I329L genes based on the TRS “GGAATATATA” has previously been used for distinguishing between closely related ASFVs [7]. The current analysis of IGR that used seven samples collected during the 2019–2022 outbreaks revealed that there were two different variants, namely, IGR II and IGR III variants of ASFV genotype 2, circulating in Can Tho domestic pig population. The IGR II variants were the most prominent (6/7 strains) and were detected in pigs in six of the provinces tested, followed by IGR III (1/7 strains) (Figure 5). The cocirculating of IGR II and III detected in the current study suggests that the spread of ASFVs was most likely caused by transportation of infected pigs from northern to southern localities [9, 12]. Interestingly, IGR III variants have never been detected in samples collected from Can Tho or any other regions in subsequent years. Therefore, it can be suggested that IGR variant II, which was initially found in northern provinces of Vietnam, spread further into almost all geographical locations of Vietnam before giving rise to variant III (in 2020–2022) when ASF occurred serially across the whole country before spreading into Can Tho city.

Altogether, this study revealed that representative ASFVs causing outbreaks in Can Tho city, a central administrative region in the Mekong delta, belonged to genotype 2 and serotype 8, and at least two variants (based on the classification of TRS in the IGR) were detected in the area. Therefore, continuous surveillance and genetic characterization are needed to facilitate sufficient understanding of local ASFV dynamics, epidemiology, and incursion of ASFV in Can Tho, Vietnam.

## Figures and Tables

**Table 1:** Details of collected samples and African swine fever viruses used in this study.

No	ASFV strains	Districts	Year	Type of sample	Flock size
1	ASF/Binh Thuy/2019	Binh Thuy	2019	Whole blood	9
2	ASF/Thoi Lai/2019	Thoi Lai	2019	Whole blood	24
3	ASF/Cai Rang/2021	Cai Rang	2021	Whole blood	15
4	ASF/Co Do/2021	Co Do	2021	Spleen, lymph node	101
5	ASF/O Mon/2021	O Mon	2021	Spleen, lymph node	22
6	ASF/Vinh Thanh/2021	Vinh Thanh	2021	Spleen, lymph node	18
7	ASF/Cai Rang/2022	Cai Rang	2022	Whole blood	125

**Table 2:** Primers used for PCR detection.

Gene	Primers	Primer's sequences (5′-3′)	Annealing temperature (°C)	Amplicon size	References
B646L	P72-U	F: GGCACAAGTTCGGACATGT	55	478 bp	[4]
	P72-D	R: GTACTGTAACGCAGCACAG			

EP402R	ASF_CD2v_ga3611-F	F: TATAATATAACAAATAATTGTAG	55	850 bp	15
	ASF_CD2v_ga4220-R	R: AGGGACGCATGTAGTAAATAG			

IGR	ASF_IGR_I73R_F	F: TGTCGTCTTACCTACAGGAT	58	651 bp	[14]
	ASF_IGR_I73R_R	R: TTCATATGCTTGTTGCGTTC			

## Data Availability

The data that support the findings of this study are available upon request from the authors.

## References

[1] Alonso C., Borca M., Dixon L. (2018). ICTV cirus taxonomy profile: *Asfarviridae*. *Journal of General Virology*.

[2] Alejo A., Matamoros T., Guerra M., Andrés G. (2018). A proteomic atlas of the African swine fever virus particle. *Journal of Virology*.

[3] Dixon L. K., Chapman D. A., Netherton C. L., Upton C. (2013). African swine fever virus replication and genomics. *Virus Research*.

[4] Bastos A. D. S., Penrith M. L., Crucière C. (2003). Genotyping field strains of African swine fever virus by partial p72 gene characterisation. *Archives of Virology*.

[5] Malogolovkin A., Burmakina G., Titov I. (2015). Comparative analysis of African swine fever virus genotypes and serogroups. *Emerging Infectious Diseases*.

[6] Quembo C. J., Jori F., Vosloo W., Heath L. (2018). Genetic characterization of African swine fever virus isolates from soft ticks at the wildlife/domestic interface in Mozambique and identification of a novel genotype. *Transboundary and Emerging Diseases*.

[7] Gallardo C., Fernández-Pinero J., Pelayo V. (2014). Genetic variation among African swine fever genotype II viruses, eastern and central Europe. *Emerging Infectious Diseases*.

[8] Le V. P., Jeong D. G., Yoon S.-W. (2019). Outbreak of african swine fever, Vietnam, 2019. *Emerging Infectious Diseases*.

[9] Nguyen V. T., Cho K.-h., Mai N. T. A. (2022). Multiple variants of African swine fever virus circulating in Vietnam. *Archives of Virology*.

[10] Tran H. T. T., Truong A. D., Dang A. K. (2021b). Genetic characterization of African swine fever viruses circulating in North Central region of Vietnam. *Transboundary and Emerging Diseases*.

[11] Tran H. T. T., Truong A. D., Ly D. V. (2020). Genetic characterisation of African swine fever virus in outbreaks in Ha Nam province, Red River delta region of Vietnam, and activity of antimicrobial products against virus infection in contaminated feed. *Journal of Veterinary Research*.

[12] Tran H. T. T., Truong A. D., Dang A. K. (2021a). Circulation of two different variants of intergenic region (IGR) located between the I73R and I329L genes of African swine fever virus strains in Vietnam. *Transboundary and Emerging Diseases*.

[13] Do D. T., Nguyen H. Q., Nguyen D. T. M., Nguyen N. M., Nguyen D. T. N., Luu H. T. Q. (2021). Genetic analysis of African swine fever virus based on major genes encoding p72, p54 and p30. *The Journal of Agriculture and Development*.

[14] Hien N. D., Nguyen L. T., Hoang L. T. (2022). First report of a complete genome sequence of a variant african swine fever virus in the Mekong delta, Vietnam. *Pathogens*.

[15] Lubisi B. A., Bastos A. D., Dwarka R. M., Vosloo W. (2018). Molecular epidemiology of African swine fever in East Africa. *Archives of Virology*.

[16] Ge S., Li J., Fan X. (2018). Molecular characterization of African swine fever virus, China, 2018. *Emerging Infectious Diseases*.

[17] Hien N. D., Hoang L. T., Phuong N. T., Tri H. M. (2021). Investigation on prevalence of African swine fever in Can Tho city. *Journal of Veterinary Science and Technology*.

